# Anxiety and depression amongst youth as adverse effects of using social media : A Review

**DOI:** 10.1097/MS9.0000000000001066

**Published:** 2023-07-07

**Authors:** Sakshi Prasad, Sara Ait Souabni, Gibson Anugwom, Kammarauche Aneni, Ayush Anand, Alexsandra Urhi, Crystal Obi-Azuike, Taneil Gibson, Asma Khan, Funso Oladunjoye

**Affiliations:** a Faculty of Medicine, National Pirogov Memorial Medical University, Vinnytsia, Ukraine; b Faculty of Medicine and Pharmacy of Marrakesh, Cadi Ayyad University, Morocco; c BP Koirala Institute of Health Sciences, Dharan, Nepal; d Mental Health Department, Federal Medical Center Asaba, Delta State, Nigeria; e Menninger Department of Psychiatry and Behavioral Sciences, Baylor College of Medicine; f Yale Child Study Center, Yale School of Medicine; g University of Medicine and Health Sciences (UMHS), New York; h Lake Erie College of Osteopathic Medicine, Pennsylvania, USA

**Keywords:** anxiety, compulsive media use, depression, fatigue, fear of missing out, social media, youth

## Abstract

Social media use has become widely popularized in modern society and because of that, human interactions have drastically changed. In parallel, depression and anxiety have reached unprecedented levels among the youth, and concerns have emerged on social media use compromising mental health. The objectives of our review are to explore if there is a relation between social media and the development of those two disorders among youth, to highlight the patterns that could lead to them, and to give recommendations for future research.

**Methods:**

Based on the Scale for the Assessment of Narrative Review Articles (SANRA) Criteria, the authors performed a search of all-time articles published in the Medline database using terms such as social media, social media use, problematic social media use, depression, anxiety, suicidality, self-harm, fear of missing out, cyberchondria, cyberbullying, sexting, and online shopping. The initial search yielded 184 924 articles. After review, 77 articles were included for discussion.

**Results:**

Social media use is often associated with depression and anxiety. Different patterns are thought to predict poorer mental health outcomes like multitasking, emotional investment, appearance-based activities, passive media use, problematic social media use, cyberbullying, sexting, and disaster awareness.

**Conclusion:**

Specific patterns of engagement with social media appear to be associated with poor mental health outcomes in youth. It is important for physicians to address social networks exposure in well-visits and for parents to communicate about it openly. However, more in-depth research needs to be done to determine a relation of causality.

## Introduction

HIGHLIGHTS

- Social media use has become widely popularized in modern society and because of that, human interactions have drastically changed.
- In parallel, depression and anxiety have reached unprecedented levels among the youth.
- Concerns have emerged on social media use compromising mental health.
- The objectives of our review are to explore if there is a relation between social media and the development of those two disorders among youth.

And, sure, fine, I do check my phone about every two minutes, but so do a lot of people, and it’s better than smoking, that’s what I say. It’s the new, lung-safe cigarette.

—Aimee Bender, The Color Master: Stories, p. 128

Social media refers to various online platforms, applications, and websites that facilitate user-generated content creation and sharing as well as communication with others^1^. The concept emerged in the 1990s with the launch of Open Diary by Bruce and Susan Abelson in 1998^1^ and Six degrees by Andrew Weinreich in 1997^2^, following widespread internet use. However, it was not until the mid-2000s when social networking achieved great success through popular platforms such as Facebook and Twitter, which redefined its essence today. Since then, numerous new platforms have arisen at a fast pace including professional networks like LinkedIn or collaborative sites such Trello while educational roles are filled by sites like Wikipedia offering valuable information for users. Social media encompasses social games, virtual worlds, and video game streaming platforms like Twitch. It influences various aspects of our lives such as online dating websites, forums, streaming blogs, and social bookmarking sites. Social media’s accessibility reaches communities worldwide and impacts interactions, thoughts, and behaviors economically and politically. As it has become mandatory in modern society replacing face-to-face encounters with texts to live video calls for human interaction, and to what will be known as the ‘Metaverse’, a project that will offer an even more interactive and immersive experience to users^3^.

Suicide rates are increasing in high-income countries among youth, particularly in girls aged 10–14. These rises are believed to be linked to social factors rather than genetics and may be influenced by the widespread use of social media platforms such as Facebook, which boasts ~2.89 billion users globally with over 90% of young adults aged between19 and 28 using it daily (and near-universal usage during the coronavirus disease 2019 (COVID-19) pandemic)^2,4–6^. Adolescence and early adulthood are critical periods of psychological development where individuals establish autonomy and independence, alongside emotional and social maturity^7^. However, these stages also pose a heightened risk for mental health issues like depression and anxiety^8,9^, especially in the current era of ‘digital natives’ who have grown up with technology as their norm^10^. The burden of anxiety disorders is significant with an estimated prevalence of 298 million cases globally in 2020 that may increase to 374 million due to COVID-19 pandemic adjustments^7^.

The prevalence of major depressive disorder is estimated at 38.7 million, rising to 49.4 million after adjusting for the COVID-19 pandemic^7^. Anxiety disorders are the most prevalent psychiatric conditions in teenagers in the United States, while depression is the most common mental health disorder in adults^8,9^. Major depressive disorder and generalized anxiety disorder represent significant financial burdens, with economic repercussions totaling US $326.2 billion by 2018 due to decreased performance and high consumption of medical resources^11–14^.

Since teenagers and young adults are the most active on social media, concerns have emerged as to whether social media use could increase their risk of mental health issues, especially depression and anxiety^2^. Many studies have been carried out in this direction, and their results are subject to discussion. Our review aims to examine whether social media use could induce depression and/or anxiety, to understand the patterns that could lead to such outcomes, and finally to suggest some recommendations and future directions in light of our findings.

## Methods

We performed our literature search based on the Scale for the Assessment of Narrative Review Articles (SANRA) Criteria [90]. On 23 December 2022, we searched the Medline database using terms such as ‘social media and depression’, ‘social media and anxiety’, ‘social media and self-harm’, ‘problematic social media use and depression’, ‘problematic social media use and anxiety’, ‘social media and suicidality’, ‘cyberchondria’, ‘fear of missing out (FOMO)’, ‘nomophobia’, ‘social media and body dissatisfaction’, ‘social gaming and depression’, ‘sexting’, and ‘cyberbullying’ and found 58 088, 57 139, 25 301, 10 494, 10 400, 8776, 418, 250, 245, 5319, 5475, 577, 2442 articles, respectively. We screened the abstracts for studies that examined social media use in relation to depression and anxiety among young people (12–26 years of age) and included the full-text studies and reviews of 77 articles that were relevant for our study objectives. All articles were cited in this article. The number of articles retrieved according to the search terms is summarized in Table 1.

**Table 1:** Search terms and results from PubMed/Medline Search

Combination search terms	Number of articles
Social media and depression	58 088
Social media and anxiety	57 139
Social media and self-harm	25 301
Problematic social media use and depression	10 494
Problematic social media use and anxiety	10 400
Social media and suicidality	8776
Cyberchondria	418
Fear of missing out FOMO	250
Nomophobia	245
Social media and body dissatisfaction	5319
Social gaming and depression	5475
Sexting	577
Cyberbullying	2442

## Results and discussion

Multiple studies have examined the duration of social media use and its impact on depression and anxiety symptoms^15–57^. Earlier studies showed a strong relation between the amount of time spent on social media and overall psychological distress^18–26^. However, recent studies suggest that the relationship between social media use and depression and/or anxiety is beyond that of frequency or duration^18–26^, so the interest has shifted toward the usage pattern. Usage pattern, whether passive (like watching content, appearance-based activities, night-time media consumption) or active (like cyberbullying and sexting), as well as emotional investment and problematic social media use (PSMU), could predict poor mental health outcomes.

### Social media use patterns and susceptible population

#### Passive social media use

Passive media use is a pattern of manipulating SM in such a way that there are no interactions with others^19^. It is the fact of simply keeping an eye on other people’s activities while keeping them out of sight and not establishing an exchange in any sort of way (not even liking content)^19^. Passive SM use among young adults can lead to a low mood and real-life interactions, ultimately leading to a sense of loneliness, a lack of confidence, self-depreciation, and jealousy^19^.

*Access to social media channels and normalizing self-harm*: There is a growing body of evidence that shows that internet users who are exposed to online self-harm content, especially visual content (images or videos), are more likely to reproduce the same behavior^20^. Also, social reinforcement on online networks has been reported to contribute significantly in aggravating self-cutting behaviors^21^. Likewise, watching these practices online motivates susceptible ones to inflict serious self-harm^22^. Teenagers are considered the most vulnerable to self-harm, and those with suicidal ideation communicate their feelings mostly to their peers than an adult or a mental health provider^23^. Also, the progression from the contemplation/ideation phase to the act or the attempt can be mediated by a certain pattern of activities on social media like reading more about suicide-related topics, commenting, and reposting suicide information^24,25^. Thus, online communication of suicidality has increased over the years^23^.

Cheng *et al.* ^26^ found that often suicide communication is done via SM platforms before suicidal attempts. In addition, live streaming suicides on social media, whether completed or failed, is a rapidly evolving practice among SM users^26^. A noteworthy example is a game called Blue Whale Challenge, which recruited players and manipulated them into committing suicide. Across the world, hundreds of teenagers from various parts of the world who were victims. Between the beginning of this phenomenon until 2017, a total of 95 555 posts related to the Blue Whale Challenge have been shared online, mainly to raise awareness about its potential dangers^27^. Despite the good intentions, they contributed to the normalization of suicidality, and aroused the curiosity of some vulnerable adolescents, leading to their copying of self-harm behaviors and the contagion effect^28^.

*Media multitasking*: Media multitasking is the fact of accessing and interacting with multiple social media platforms at the same time. This new trend has grown bigger among internet users and is turning into the new normal as to how one uses social media. Becker *et al.* ^29^ demonstrated that media multitasking was associated with higher depression and social anxiety rates, even when controlling personality traits and overall media use. Moreover, multitasking negatively impacts productivity, task completion, performance, and attention^30^, which might contribute to feelings of guilt and worthlessness^30^.

*Appearance-based social media activities and social comparisons*: Increasingly high use of social media filters that alter facial features and retouches body parts may contribute to poor mental health outcomes. In addition, there are also lighting hacks, photo, and video editing used by most consumers and influencers before posting their content. The continuous exposure to these photographs and recordings leads to a distorted image of the world and of reality and gives the illusion that people have picture-perfect bodies and flawless features^31^. Hawes *et al.* ^31^ suggested that the appearance-based activities can lead to psychological distress due to judging one’s physique, body dissatisfaction, eating disorders, depression, and anxiety disorders.

*Night-time media consumption*: Smartphone use during the night and mostly before bed has become a habit for many people, especially teenagers, and has been linked to sleep deprivation and a decrease in sleep quality^32^. Nevertheless, night-time media consumption and its relation to depression and anxiety have not been deeply investigated^32^. A study by Lemola *et al.* pointed towards a relation between night-time SM use and depression. Also, Woods and Scott^32^ highlighted that night-time-specific social media use could indeed predict depression and anxiety; however, emotional investment was a stronger factor.

*Disaster awareness*: These days online newspapers actively utilize SM to reach a bigger audience. The SM algorithms are done in such a way that it sorts users’ preferences and expose them consistently to the similar content. Reading about natural and social disasters, wars and conflicts, terrorism, and pandemics, makes users indirectly prone to stress^33^ and exacerbates already pre-existing anxiety. Some studies have suggested that there is a correlation between the spreading of infectious diseases and levels of anxiety and depression^33–35^. For example, several studies highlighted how COVID-19 anxiety and depression-related symptoms were aggravated by SM use^35^.

#### Active social media use

Active social media use is defined as “online behaviors that facilitate ‘direct exchanges’ among users^19^”. Such behaviors include messaging, liking, sharing, or posting content. Although some studies demonstrated that being active on social media was negatively associated with depression^19,36^, some patterns, particularly sexting and cyberbullying, can be related to depression and anxiety.

*Sexting*: Sexting means electronically sending sexually suggestive texts, images, or videos to another person^37^. Sexting can be considered as a normal contemporary way of showing intimacy^38^. However, its consequences can be serious, especially when there is unwanted dissemination, harassment, or blackmail. There are disparities in results regarding sexting and its effect on mental health^39,40^, which may have to do something with the method of sexting. There are two ways of sexting: active (when the person actively sends revealing content) or passive (when the person only receives it)^40^, which can be consensual or coerced. Gass *et al.* showed that there was a substantial difference between psychopathology due to sexting between young adult males and females. Though men were not affected by benevolent sexting, women were at higher risk of developing anxiety and depression even with passive sexting. On the other hand, both sexes develop poorer mental health when there is online sexual victimization^41^, as the receipt of unwanted sexts, oppression, or coercion^42^. Demographic variables may also explain the discrepancy between the results. Wachs *et al*.^43^ reported that teenagers engaged in sexting, particularly girls and nonminority teens were at risk of experiencing depressive symptoms and self-harm. Moreover, the media have pointed out several suicide stories among teenagers that were humiliated and bullied after their photos/videos spread everywhere on the internet without their consent. In the same way, sexters can fall prey to blackmail, which could lead to both anxiety and depression.

*Cyberbullying and online ethnic discrimination*: Cyberbullying, is a type of bullying that is conducted through online networks, and it can take much higher dimensions compared to traditional bullying and is more likely to lead to worse outcomes^45^. The lack of accountability on behalf of social media service providers can make the situation worse. The automated moderating tools of social media service providers often fail to erase intimidating or insulting comments, and it is hard to sue someone whose identity is unknown. Furthermore, rapid and macro-scale dissemination of sensitive content without restriction can be a major challenge to deal with. That being said, there are few studies that examine these aspects concretely. Hellfeldt *et al*.^45^ suggested that depression and anxiety symptoms were significantly high in both the victims of cyberbullying (cyber-victims) and in people who are bullied, whilst bullying others at the same time (cyber-bully victims). Female victims are more susceptible to developing depression, substance use disorder, and suicidal ideation as compared to male victims^46^. In the same way, Cano *et al*.^47^ demonstrated that young adults that undergo ethnic and racial discrimination via social media are more prone to developing depression and anxiety. On the other hand, parental, and social support^46,47^, as well as, growth mindset^48^ seem to be protective factors.

*Emotional investment*: Alsunni and Latif^49^ demonstrated that emotional investment was one of the most significant factors leading to depression, anxiety, and a lack of self-esteem among social media customers. Other studies also found emotional components, among other factors, such as night-time media consumption and the amount of time spent on social networks, to contribute to the development of depression and anxiety among excessive social media users^50,51^. These results might be explained by ‘The dualistic model of passion’, which has been successfully transposed to internet use^49,52^. This model depicts two representations of ‘passion’. The ‘harmonious passion’ leads to adaptive behavior, and the ‘obsessive passion’, leads to compulsive behavior and utterly hurts well-being^52^. When obsessive passion is developed using social media, it might theoretically lead to mental health issues.

*Problematic social media use and its risk factors*: PSMU is described as the irresistible urge to use or dependence on social media^45^. It can result in psychological, physical, academic, and social distress of the affected individual^45,46^. Currently, DSM V does not place it under the scope of addictions, although it has been put forward. PSMU can be a predictor of depression and anxiety among internet users, and can be associated with feelings of unhappiness, negative affect, a lack of self-esteem^47,48^, and stress-related symptoms^48,49^. Social belongingness and the need for social assurance serve as a remarkable mediator, linking social anxiety to PSMU^50^. Moreover, in many cases, PSMU coexists with other addictive behaviors like alcohol and cannabis addictions^51^, together with certain personality disorders, namely Clusters A and B, as well as some antisocial personality traits, namely Machiavellianism, narcissism, psychopathy, and sadism^52^. Not enough studies have been carried out regarding the following subjects but have been implicated to predict the susceptibility to develop depressive and/or anxiety disorders:

*Social gaming*: Social gaming is the act of playing online video games with other people simultaneously. It has been made possible by the development of multiplayer modes and the addition of communication tools within the games (chat boxes, calling, and video calling options), allowing collaboration to complete tasks or competition to win. Internet gaming disorder has been integrated into DSM V psychological disorders, as well as the international classification of diseases^53^, but to date, we do not have a clear subcategorization^54^. More studies should be directed towards social gaming in relation to depression and anxiety, as there is a staggering paucity of papers on that subject and the few studies that were made lack reliability.

*Accessing nudity via social media*: SM is increasingly used for advertising nudity and pornographic content. ‘Only fans’ is the most known platform serving that purpose. One can pay a monthly or yearly subscription to see photos/videos of the creator of their choice, whether it is a sex worker, a fitness expert, an influencer, a musician, or an amateur who decides to indulge in this activity^55^. As of 2019, a new option of going live has been added. Accessing pornography on internet websites is a customary practice nowadays, and perceived pornography addiction is positively correlated to neuroticism, psychological distress, and anxiety^55^; but is this behavior increased by social media? We could not find studies to answer that.

*Enhancing online shopping*. It is well-known that social media is one of the most effective advertising platforms. The numbers of adverts have increased dramatically, and more companies and even small businesses now turn to digital marketing, as a new, effective, low-cost, and easy alternative for promoting their products. As a result, online shopping is thriving. However, not enough studies have examined the relation between SMU with excessive buying and their possible mediating role in developing depression and anxiety among compulsive buyers^56^.

*Social Media and Cyberchondria*: The internet is a tool used by everyone to address apprehensions and answer questions. Sometimes these concerns are about medical issues or symptoms. From many resources, we know that people use the internet to look for information regarding their health^57^. Forums that discuss health issues are a way of getting information, and oftentimes people post their symptoms on social media to have others’ opinions. This behavior could theoretically be a mediator for ‘cyberchondria’^57^ which is an anxiety that is developed by looking excessively for one’s symptoms on the internet; however, there is not enough information on the subject.

### Evidence from experimental studies

Though there are detrimental effects of SM use among youth, it can also be used for intervention related to mental health issues. Radovic *et al.* conducted a pilot randomized controlled trial among adolescents with anxiety or depression requiring treatment to assess the utility of a peer support website intervention, and found that a website-led intervention led to a statistically significant treatment uptake in the intervention group than the usual care group. Another randomized controlled trial by McCall *et al.* among university students reported a significantly lower mean social anxiety scale score in the website-based intervention group than in the usual care group, with approximately four-fifths of the participants in the intervention group reporting satisfactory care^58^. Another study by Amon *et al.* ^59^ studied the efficacy of online counseling of young people (13–25 years of age) for mental health problems using a social networking site, and revealed a significant reduction in anxiety and depression at the midpoint of intervention and a more substantial decrease in symptoms at the end. In addition to these studies, a qualitative study was conducted by Gewali *et al.* ^60^ to understand the feasibility of a social media-based cognitive behavioral therapy to prevent perinatal depression among young adults. They concluded that social media-based intervention could help address depression among young adults and should be used to foster evidence-based intervention for depression.

The recent COVID-19 pandemic led to a rise in SM use as people were locked in their homes. Though the detrimental effects of SM use have been dealt in details earlier, the SM platforms were also used for successful interventions for mental health issues. During the COVID-19 pandemic, Schleider *et al.* conducted a randomized controlled trial to assess the efficacy of a single online-based intervention (SSIs) for depression among adolescents. The effectiveness of growth mindset SSI (GM-SSI) and behavioral action SSI (BA-SSI) was compared with the placebo group using various standardized scales. Both SSIs were acceptable to the majority of participants^61^. There was a significant decrease in depression symptoms following the intervention and at the three-month follow-up^61^. Also, the reduction in depressive symptoms was more for the BA-SSI group than the GM-SSI group at follow^61^.

Overall, these studies showed that social media interventions could significantly decrease the severity of anxiety and depression in adolescents and young adults. In addition, social media-based interventions are also acceptable to the majority of adolescents and young adults. Hence, the evidence-based application of these interventions can prove to be a crucial strategy for treating depression and anxiety.

#### Clinical implications

‘The media are not the leading cause of any health problem in childhood or adolescence. However, they can make a substantial contribution to virtually every health concern that pediatricians and parents have about young people—aggression, sex, drugs, obesity, self-image and eating disorders, depression, and suicide, even learning disorders and academic achievement’. Dr Victor Strasburger^62^.

With the rapidly changing social media interface now, most of the recommendations would be preliminary. Despite this shortcoming of a limited evidence base at this time, it is best advised by studies to use a decision tree approach^63^ to mediate digital network usage amongst adolescents. Different sets of bodies can work synergistically in helping adolescents make the changes needed in their use of social media, and in modifying or changing their attitudes towards social media usage^64^.

1. Clinicians treating young adults with mental distress, or those at the risk of developing such conditions can discuss with young adults and their parents the known risks of social media usage.
2. Pediatricians and clinicians should screen for media exposure (quality, duration, and frequency) to help reduce symptoms potentially indicative of exposure to problematic media and treat patients suffering from symptoms of anxiety, low self-esteem, insomnia, etc.
3. Reducing the time spent on social media and television as part of a harm reduction approach should be advocated for by stakeholders in child health, like child psychiatrists and other mental health professionals who work with young adults. Prolonged exposure has been shown to increase one’s chances of developing depression^65^. Complete abstinence is not suggested as well by studies^65,66^. Likewise, a study by Kushlev *et al.* ^67^, suggests poorer quality interactions with children amongst parents with heavy smartphone use. It is worthwhile reminding parents to model their own smartphone use.
4. Reduced levels of depressive and anxiety symptoms were reported in young adults belonging to households that had implemented rules regarding media use according to a study^68^ and similar rules can be implemented by parents for regulated use of social media. For instance, a basic recommendation could be made to remove smartphones from the bedroom, to improve sleep hygiene.
5. A study by Kim and White^69^, suggested that while parental limits may be useful in reducing social media use, open and nonjudgmental communication, eliciting trust and emotional safety, and offering a sense of independence and autonomy can prove to be a more effective method of the therapeutic alliance with youth.
6. Pediatricians can also guide parents to help children consume age-appropriate content with the help of ratings, reviews, plot descriptions, and by screening content before their child has access to it while being considerate of their age, adolescence, and changing needs.
7. For the group of children and adolescents whose doctors feel they may be vulnerable to social anxiety disorder, children could be encouraged to take part in many opportunities to practice face-to-face communication (school activities involving competitions) and to limit online communication.
8. Policymakers should advocate for the development of more accurate, helpful application ratings and blocking technologies that raise suspicion or increase the potential risk of psychological disorders and enforce their proper application.
9. The American Academy of Pediatrics provides an elaborate guideline to support youth regarding the use of social media^70^. Further, the American Academy of Pediatrics has partnered with an organization called ‘Common Sense Media’ to produce a media toolkit containing useful information for parents^71^. Another organization developed by former technology industry members is ‘Center for Humane Technology (http://humanetech.com/), developed out of concern and provides practical strategies to curb the potentially deleterious effects of new media on psychological states.
10. At the system level, school, and community-based limits on social media use have been implemented in several jurisdictions, with mixed results^72^. Such interventions should respectfully ensure adolescents’ autonomy and be developmentally appropriate. Rather than a complete ban on electronics and social media within school premises, a more productive approach shall be a negotiation between teacher and pupils, as developmentally appropriate, and with mutual trust and respect for autonomy^73,74^.
11. A qualitative study by Royen *et al.* ^75^, found young adults valuing their freedom and privacy but also recognizing the need for cybersecurity and automatic monitoring in situations beyond an individual’s control. Young adults have started realizing the potential side effects social media can pose in their lives and are taking steps to mitigate this^66^.

#### Call for further research

1. A significant amount of past research about how social media and social networking affect anxiety, depression, and related mental well-being are cross-sectional studies and hence causality cannot be determined^70^. For instance, most of our questions asked above like ‘does social media induce these mood disorders’, or ‘does it only exacerbate already pre-existing symptoms’, or ‘is smartphone addiction inherent in its connections to offline society, such as offline social networks and social engagement’. Future research should clarify whether compulsive social media use has a long-term effect on individuals and to what extent^76^ and aid in drawing a clearer picture of the connection between social networking and how this relationship might change or be reinforced over time.
2. Along with aiding in diagnosing these digitally induced psychological disorders, future research should examine the reason some children lack social resources, and the role interventions can play and help focus on expanding support networks to reduce the incidence of these preventable disorders.
3. Future studies can also evaluate in more detail the understanding of parents’/ guardians’ and pediatrician’s perception of children’s social networking behaviors, and online social engagement, which may help provide a more concrete understanding of social media and its impact on adolescents.

## Conclusion

Social media use contributes to depression and anxiety among youth. However, the patterns by which this occurs remain to be elucidated. Several factors appear to be relevant including activities done through social media such as bullying, false appearances, or through content imbibed from watching things on social media which may be unhelpful. Understanding the pathways through which social media use contributes to depression and anxiety will inform specific youth-centered interventions.

## Ethical approval and consent to participate

Not applicable.

## Consent for publication

Received from all authors.

Patient Consent - Not applicable.

## Sources of funding

All authors have declared that no financial support was received from any organization for the submitted work. All authors have declared that there are no other relationships or activities that could appear to have influenced the submitted work.

## Author contribution

S.P., S.A.S., and G.A. were involved with the primary conception of the idea and writing the first draft; K.A., A.U., and C.A. were involved in including the number and kind of studies and deciding the criteria of studies to be included; A.K. and T.G. helped in supervision and final edit of the draft along with F.O.

## Conflicts of interest disclosure

All authors have declared that they have no financial relationships at present or within the previous three years with any organizations that might have an interest in the submitted work.

## Research registration unique identifying number (UIN)

1. Name of the registry: not applicable.
2. Unique Identifying number or registration ID: not applicable.
3. Hyperlink to your specific registration (must be publicly accessible and will be checked): not applicable.

## Guarantor

Sakshi Prasad.

## Availability of data and material

Not applicable.

## Provenance and peer review

Not commissioned, externally peer-reviewed.

## References

[1] Kaplan AM Haenlein M. Users of the world, unite! The challenges and opportunities of Social Media. Bus Horiz 2010;53:59–68.

[2] Primack BA Escobar-Viera CG. Social media as it interfaces with psychosocial development and mental illness in transitional age youth. Child Adolesc Psychiatr Clin N Am 2017;26:217–233. 28314452 10.1016/j.chc.2016.12.007

[3] Siyaev A Jo GS. Towards aircraft maintenance metaverse using speech interactions with virtual objects in mixed reality. Sensors 2021;21:2066. 33804253 10.3390/s21062066 PMC8001242

[4] Padmanathan P Bould H Winstone L. Social media use, economic recession and income inequality in relation to trends in youth suicide in high-income countries: a time trends analysis. J Affec Dis 2020;275:58–65. 10.1016/j.jad.2020.05.057 PMC7397515 32658824

[5] Luby J Kertz S. Increasing suicide rates in early adolescent girls in the united states and the equalization of sex disparity in suicide: the need to investigate the role of social media. JAMA Netw Open 2019;2:e193916. 31099856 10.1001/jamanetworkopen.2019.3916

[6] Pew Research Center. Social media update 2021. 2021. Accessed 17 December 2021. https://www.pewresearch.org/internet/2021/04/07/social-media-use-in-2021/

[7] COVID-19 Mental Disorders Collaborators. Global prevalence and burden of depressive and anxiety disorders in 204 countries and territories in 2020 due to the COVID-19 pandemic. Lancet 2021;398:1700–1712. 34634250 10.1016/S0140-6736(21)02143-7 PMC8500697

[8] Merikangas KR He JP Burstein M. Lifetime prevalence of mental disorders in U.S. adolescents: results from the National Comorbidity Survey Replication–Adolescent Supplement (NCS-A). J Am Acad Child Adolesc Psychiatry 2010;49:980–989. 20855043 10.1016/j.jaac.2010.05.017 PMC2946114

[9] Kessler RC Berglund P Demler O. Lifetime Prevalence and Age-of-Onset Distributions of DSM-IV Disorders in the National Comorbidity Survey Replication. Arch Gen Psychiatry 2005;62:593. 15939837 10.1001/archpsyc.62.6.593

[10] Crone EA Konijn EA. Media use and brain development during adolescence. Nat Commun 2018;9:588; Published 2018 Feb 21. 29467362 10.1038/s41467-018-03126-x PMC5821838

[11] Greenberg PE Fournier AA Sisitsky T. The economic burden of adults with major depressive disorder in the United States (2010 and 2018). Pharmacoeconomics 2021;39:653–665. 33950419 10.1007/s40273-021-01019-4 PMC8097130

[12] Greenberg PE Fournier AA Sisitsky T. The economic burden of adults with major depressive disorder in the United States (2005 and 2010). J Clin Psychiatry 2015;76:155–162. 25742202 10.4088/JCP.14m09298

[13] Briley M Lépine. The increasing burden of depression. NDT 2011;7:3; Published online. 10.2147/NDT.S19617 PMC3131101 21750622

[14] Revicki DA Travers K Wyrwich KW. Humanistic and economic burden of generalized anxiety disorder in North America and Europe. J Affec Dis 2012;140:103–112. 10.1016/j.jad.2011.11.014 22154706

[15] Hunt MG Marx R Lipson C. No more FOMO: limiting social media decreases loneliness and depression. J Soc Clin Psychol 2018;37:751–768.

[16] Costa RM Patrão I Machado M. Problematic internet use and feelings of loneliness. Int J Psych Clin Prac 2019;23:160–162. 10.1080/13651501.2018.1539180 30570343

[17] Bányai F Zsila Á Király O. Problematic social media use: results from a large-scale nationally representative adolescent sample. PLoS One 2017;12:e0169839; Published 2017 Jan 9. 28068404 10.1371/journal.pone.0169839 PMC5222338

[18] Gupta M Sharma A. Fear of missing out: a brief overview of origin, theoretical underpinnings and relationship with mental health. WJCC 2021;9:4881–4889. 34307542 10.12998/wjcc.v9.i19.4881 PMC8283615

[19] Trifiro BM Gerson J. Social media usage patterns: research note regarding the lack of universal validated measures for active and passive use. Soc Med + Soc 2019;5:205630511984874.

[20] Daine K Hawton K Singaravelu V. The power of the web: a systematic review of studies of the influence of the internet on self-harm and suicide in young people. PLoS ONE 2013;8:e77555; García AV, ed. 24204868 10.1371/journal.pone.0077555 PMC3813687

[21] Brown RC Fischer T Goldwich AD. #cutting: Non-suicidal self-injury (NSSI) on Instagram. Psychol Med 2018;48:337–346. 28705261 10.1017/S0033291717001751

[22] Jacob N Evans R Scourfield J. The influence of online images on self-harm: a qualitative study of young people aged 16–24. J Adol 2017;60:140–147. 10.1016/j.adolescence.2017.08.001 PMC5614108 28881214

[23] L. Belfort E Mezzacappa E Ginnis K. Similarities and differences among adolescents who communicate suicidality to others via electronic versus other means: a pilot study. APS 2012;2:258–262.

[24] Liu X Huang J Yu NX. Mediation effect of suicide-related social media use behaviors on the association between suicidal ideation and suicide attempt: cross-sectional questionnaire study. J Med Internet Res 2020;22:e14940. 32343249 10.2196/14940 PMC7218592

[25] Cero I Witte TK. Assortativity of suicide-related posting on social media. American Psychologist 2020;75:365–379. 31192621 10.1037/amp0000477 PMC7061921

[26] Cheng Q Kwok C Zhu T. Suicide Communication on Social Media and Its Psychological Mechanisms: An Examination of Chinese Microblog Users. IJERPH 2015;12:11506–11527. 26378566 10.3390/ijerph120911506 PMC4586688

[27] Cheng Q Kwok C Zhu T. Suicide communication on social media and its psychological mechanisms: an examination of Chinese microblog users. IJERPH 2015;12:11506–11527. 26378566 10.3390/ijerph120911506 PMC4586688

[28] Khasawneh A Chalil Madathil K Dixon E. Examining the self-harm and suicide contagion effects of the blue whale challenge on YouTube and Twitter: qualitative study. JMIR Ment Health 2020;7:e15973; Published 2020 Jun 5. 32515741 10.2196/15973 PMC7312265

[29] Becker MW Alzahabi R Hopwood CJ. Media multitasking is associated with symptoms of depression and social anxiety. Cyberpsychol Behav Soc Net 2013;16:132–135. 10.1089/cyber.2012.0291 23126438

[30] Appelbaum SH Marchionni A Fernandez A. The multi‐tasking paradox: perceptions, problems and strategies. Manag Dec 2008;46:1313–1325.

[31] Hawes T Zimmer-Gembeck MJ Campbell SM. Unique associations of social media use and online appearance preoccupation with depression, anxiety, and appearance rejection sensitivity. Body Image 2020;33:66–76. 32113009 10.1016/j.bodyim.2020.02.010

[32] Woods HC Scott H. #Sleepyteens: social media use in adolescence is associated with poor sleep quality, anxiety, depression and low self-esteem. J Adol 2016;51:41–49. 10.1016/j.adolescence.2016.05.008 27294324

[33] Lee E Lee H. Disaster awareness and coping: impact on stress, anxiety, and depression. Perspect Psychiatr Care 2019;55:311–318. 30648274 10.1111/ppc.12351

[34] Witthauer C Gloster AT Meyer AH. Comorbidity of infectious diseases and anxiety disorders in adults and its association with quality of life: a community study. Front Public Health 2014;2:80. 25072049 10.3389/fpubh.2014.00080 PMC4095564

[35] Twenge JM Campbell WK. Media use is linked to lower psychological well-being: evidence from three datasets. Psychiatr Q 2019;90:311–331. 30859387 10.1007/s11126-019-09630-7

[36] Escobar-Viera CG Shensa A Bowman ND. Passive and active social media use and depressive symptoms among united states adults. Cyberpsychology, Behavior, and Social Networking 2018;21:437–443. 29995530 10.1089/cyber.2017.0668

[37] Temple JR Le VD van den Berg P. Brief report: teen sexting and psychosocial health. J Adol 2014;37:33–36. 10.1016/j.adolescence.2013.10.008 PMC3896072 24331302

[38] Döring N. Consensual sexting among adolescents: Risk prevention through abstinence education or safer sexting? Cyberpsychol Psychosocial ResCyberspace 2014;8. 10.5817/CP2014-1-9

[39] Gordon-Messer D Bauermeister JA Grodzinski A. Sexting among young adults. J Adol Health 2013;52:301–306. 10.1016/j.jadohealth.2012.05.013 PMC3580013 23299018

[40] Gassó K Agustina M. Sexting, mental health, and victimization among adolescents: a literature review. IJERPH 2019;16:2364. 31277335 10.3390/ijerph16132364 PMC6650829

[41] Gassó AM Mueller-Johnson K Montiel I. Sexting, online sexual victimization, and psychopathology correlates by sex: depression, anxiety, and global psychopathology. IJERPH 2020;17:1018. 32041115 10.3390/ijerph17031018 PMC7036947

[42] Klettke B Hallford DJ Clancy E. Sexting and psychological distress: the role of unwanted and coerced sexts. Cyberpsychol, Behav Soc Network 2019;22:237–242. 10.1089/cyber.2018.0291 30855187

[43] Wachs S Wright MF Gámez-Guadix M. How Are consensual, non-consensual, and pressured sexting linked to depression and self-harm? the moderating effects of demographic variables. IJERPH 2021;18:2597. 33807667 10.3390/ijerph18052597 PMC7967514

[44] Moore SE Norman RE Suetani S. Consequences of bullying victimization in childhood and adolescence: a systematic review and meta-analysis. World J Psychiatry 2017;7:60–76. 28401049 10.5498/wjp.v7.i1.60 PMC5371173

[45] Hellfeldt K López-Romero L Andershed H. Cyberbullying and psychological well-being in young adolescence: the potential protective mediation effects of social support from family, friends, and teachers. IJERPH 2019;17:45. 31861641 10.3390/ijerph17010045 PMC6981789

[46] Niu G He J Lin S. Cyberbullying victimization and adolescent depression: the mediating role of psychological security and the moderating role of growth mindset. IJERPH 2020;17:4368. 32570765 10.3390/ijerph17124368 PMC7345096

[47] Cano MÁ Schwartz SJ MacKinnon DP. Exposure to ethnic discrimination in social media and symptoms of anxiety and depression among Hispanic emerging adults: Examining the moderating role of gender. J Clin Psychol. 2021;77:571–586. 32869867 10.1002/jclp.23050 PMC7878314

[48] Cano MÁ Schwartz SJ MacKinnon DP. Exposure to ethnic discrimination in social media and symptoms of anxiety and depression among Hispanic emerging adults: Examining the moderating role of gender. J Clin Psychol 2021;77:571–586. 32869867 10.1002/jclp.23050 PMC7878314

[49] Alsunni AA Latif R. Higher emotional investment in social media is related to anxiety and depression in university students. J Taibah Univ Med Sci 2021;16:247–252. 33897330 10.1016/j.jtumed.2020.11.004 PMC8046824

[50] Shensa A Sidani JE Dew MA. Social media use and depression and anxiety symptoms: a cluster analysis. Am J Health Behav 2018;42:116–128. 29458520 10.5993/AJHB.42.2.11 PMC5904786

[51] Lin LY Sidani JE Shensa A. Association between social media use and depression among u.s. young adults: research article: social media and depression. Depress Anx 2016;33:323–331. 10.1002/da.22466 PMC4853817 26783723

[52] Vallerand RJ Liu WC Wang JCK Ryan RM. The Dualistic Model of Passion: Theory, Research, and Implications for the Field of Education. Building Autonomous Learners. Springer; 2016. 31-58. doi:10.1007/978-981-287-630-0_3

[53] The Lancet. ICD-11. Lancet 2019;393:2275. 31180012 10.1016/S0140-6736(19)31205-X

[54] American Psychiatric Association. Diagnostic and Statistical Manual of Mental Disorders Fifth Edition. American Psychiatric Association; 2013. doi:10.1176/appi.books.9780890425596

[55] Grubbs JB Stauner N Exline JJ. Perceived addiction to Internet pornography and psychological distress: examining relationships concurrently and over time. Psychol Add Behav 2015;29:1056–1067. 10.1037/adb0000114 26372200

[56] Tian Y Zhang S Wu R. Association between specific internet activities and life satisfaction: the mediating effects of loneliness and depression. Front Psychol 2018;9:1181. 30050484 10.3389/fpsyg.2018.01181 PMC6050461

[57] Seyed Hashemi SG Hosseinnezhad S Dini S. The mediating effect of the cyberchondria and anxiety sensitivity in the association between problematic internet use, metacognition beliefs, and fear of COVID-19 among Iranian online population. Heliyon 2020;6:e05135. 33072911 10.1016/j.heliyon.2020.e05135 PMC7547399

[58] McCall HC Richardson CG Helgadottir FD. Evaluating a web-based social anxiety intervention among university students: randomized controlled trial. J Med Internet Res 2018;20:e91. 29563078 10.2196/jmir.8630 PMC5885061

[59] Amon K Ridout B Forsyth R. Online group counseling for young people through a customized social networking platform: phase 2 of kids helpline circles. Cyberpsychol Behav Soc Network 2022;25:580–588. 10.1089/cyber.2022.0044 35951017

[60] Gewali A Lopez A Dachelet K. A social media group cognitive behavioral therapy intervention to prevent depression in perinatal youth: stakeholder interviews and intervention design. JMIR Ment Health 2021;8:e26188. 34524086 10.2196/26188 PMC8482173

[61] Schleider JL Mullarkey MC Fox KR. A randomized trial of online single-session interventions for adolescent depression during COVID-19. Nat Hum Behav 2022;6:258–268. 34887544 10.1038/s41562-021-01235-0 PMC8881339

[62] Strasburger VC Jordan AB Donnerstein E. Health effects of media on children and adolescents. Pediatrics 2010;125:756–767. 20194281 10.1542/peds.2009-2563

[63] Lakasing E Mirza Z. Anxiety and depression in young adults and adolescents. Br J Gen Pract 2020;70:56–57. 32001455 10.3399/bjgp20X707765 PMC7018423

[64] Hoge E Bickham D Cantor J. Digital media, anxiety, and depression in children. Pediatrics 2017;140(Suppl 2):S76–S80. 29093037 10.1542/peds.2016-1758G

[65] Primack BA Swanier B Georgiopoulos AM. Association between media use in adolescence and depression in young adulthood: a longitudinal study. Arch Gen Psychiatry 2009;66:181–188. 19188540 10.1001/archgenpsychiatry.2008.532 PMC3004674

[66] Abi-Jaoude E Naylor KT Pignatiello A. Smartphones, social media use and youth mental health. CMAJ 2020;192:E136–E141. 32041697 10.1503/cmaj.190434 PMC7012622

[67] Kushlev K Dunn EW. Smartphones distract parents from cultivating feelings of connection when spending time with their children. J Soc Pers Relat 2019;36:1619–1639.

[68] Bickham DS Hswen Y Rich M. Media use and depression: exposure, household rules, and symptoms among young adolescents in the USA. Int J Public Health 2015;60:147–155. 25586816 10.1007/s00038-014-0647-6 PMC4375733

[69] Kim B White K. How can health professionals enhance interpersonal communication with adolescents and young adults to improve health care outcomes?: systematic literature review. Int J Adolesc Youth 2018;23:198–218.

[70] Children and media tips from the American Academy of Pediatrics. Itasca (IL): American Academy of Pediatrics; 2018. Accessed 11 February 2022. https://www.aap.org/en-us/about-the-aap/aap-press-room/news-features-and-safety-tips/Pages/Children-and-Media -Tips.aspx

[71] Family Media Toolkit: age-based guidelines for children’s media and device use. San Francisco: Common Sense. Accessed 11 February 2022. https://www.commonsensemedia.org/ AAPtoolkit

[72] Beland L-P Murphy R. Ill Communication: technology, distraction & student performance. Labour Econ 2016;41:61–76.

[73] Charles AS Michele Knobel Colin Lankshear Michele Knobel Colin Lankshear Chapter eleven: “There’s a relationship”: Negotiating cell phone use in the high school classroom. Researching New Literacies: Design, Theory, and Data in Sociocultural Investigation: Peter Lang, International Academic Publishers; 2017. Accessed 14 October 2019. https://www.peterlang.com/view/9781433138331 /xhtml/chapter11.xhtml

[74] Tatum NT Olson MK Frey TK. Noncompliance and dissent with cell phone policies: a psychological reactance theoretical perspective. Commun Educ 2018;67:226–244.

[75] Van Royen K Poels K Vandebosch H. Harmonizing freedom and protection: Adolescents’ voices on automatic monitoring of social networking sites. Child Youth Serv Rev 2016;64:35–41.

[76] Pera A. The psychology of addictive smartphone behavior in young adults: problematic use, social anxiety, and depressive stress. Front Psychiatry 2020;11:573473. 33101087 10.3389/fpsyt.2020.573473 PMC7522217

